# Recent Advances on 3D-Printed Zirconia-Based Dental Materials: A Review

**DOI:** 10.3390/ma16051860

**Published:** 2023-02-24

**Authors:** Ana Catarina Branco, Rogério Colaço, Célio Gabriel Figueiredo-Pina, Ana Paula Serro

**Affiliations:** 1 Centro de Química Estrutural (CQE), Departamento de Engenharia Química, Institute of Molecular Sciences, Instituto Superior Técnico, Universidade de Lisboa, Av. Rovisco Pais, 1049-001 Lisbon, Portugal; 2 Centro de Desenvolvimento de Produto e Transferência de Tecnologia, Department of Mechanical Engineering, Escola Superior de Tecnologia de Setúbal, Instituto Politécnico de Setúbal, Estefanilha, 2910-761 Setúbal, Portugal; 3 Centro de Investigação Interdisciplinar Egas Moniz, Instituto Universitário Egas Moniz, Quinta da Granja, Monte de Caparica, 2829-511 Caparica, Portugal; 4 Departamento de Engenharia Mecânica, Instituto de Engenharia Mecânica, Instituto Superior Técnico, Universidade de Lisboa, 1049-001 Lisboa, Portugal; 5 Center of Physics and Engineering of Advanced Materials, Instituto Superior Técnico, University of Lisbon, Av. Rovisco Pais, 1049-001 Lisboa, Portugal

**Keywords:** additive manufacturing, 3D printing, dental materials, ceramic materials, zirconia, dental applications

## Abstract

Zirconia-based materials are widely used in dentistry due to their biocompatibility and suitable mechanical and tribological behavior. Although commonly processed by subtractive manufacturing (SM), alternative techniques are being explored to reduce material waste, energy consumption and production time. 3D printing has received increasing interest for this purpose. This systematic review intends to gather information on the state of the art of additive manufacturing (AM) of zirconia-based materials for dental applications. As far as the authors know, this is the first time that a comparative analysis of these materials’ properties has been performed. It was performed following the PRISMA guidelines and using PubMed, Scopus and Web of Science databases to select studies that met the defined criteria without restrictions on publication year. Stereolithography (SLA) and digital light processing (DLP) were the techniques most focused on in the literature and the ones that led to most promising outcomes. However, other techniques, such as robocasting (RC) and material jetting (MJ), have also led to good results. In all cases, the main concerns are centered on dimensional accuracy, resolution, and insufficient mechanical strength of the pieces. Despite the struggles inherent to the different 3D printing techniques, the commitment to adapt materials, procedures and workflows to these digital technologies is remarkable. Overall, the research on this topic can be seen as a disruptive technological progress with a wide range of application possibilities.

## 1. Introduction

**Figure 1:**
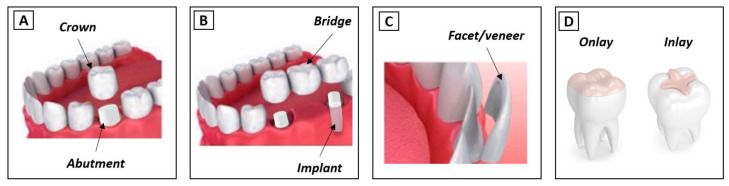
Types of dental restorations (adapted from [2]). (**A**) Crown and abutment; (**B**) Bridge and implant; (**C**) Facet/veneer; (**D**) Onlay and inlay.

Teeth damage/loss has strong implications in phonetics, aesthetics, and mastication processes [1]. The repair/replacement of the damaged/lost tissues is carried out using artificial materials, which should be able to withstand the severe mechanical, chemical and thermal oral requirements. Currently, there are different materials and techniques that allow researchers to restore the function of dental tissues. Ceramic materials are quite widely used for this purpose, mainly due to their aesthetic properties. However, they raise concerns related to the risk of failure induced by fatigue processes that decrease their ability to support the high loads endured during biting and mastication. Furthermore, the wear induced on the antagonist dental surface, mainly due to microfracture-based wear mechanisms, is also an issue [2]. Ceramics used in dentistry can be divided into two main groups: low- and high-toughness ceramics. The first type corresponds to vitroceramic materials, such as leucite or lithium disilicate, and is essentially used in the restoration of dental crowns (inlays/onlays, facets and veneers—see Figure 1C,D). However, the brittle behavior of these materials impairs their performance. They suffer wear, which is generally associated with fracture/chipping wear mechanisms. The formation of third-body particles dramatically increases dental wear. Contrarily, in high-toughness ceramics such as zirconia, the prosthetic material wear is negligible, and the wear on the opponent teeth is much lower than that observed against vitroceramic materials. In this case, the main wear mechanism of the teeth results from the penetration of ceramic harder-surface asperities in the enamel/dentine that cut/plough these softer tissues (two-body abrasion) [2,3,4]. Thus, in this case, the wear is highly affected by the prosthetic material surface roughness [5]. In dentistry, zirconia is commonly applied in the production of dental crowns, bridges, implants and abutments (Figure 1A,B). More, this material can be used to substitute or repair both anterior and posterior teeth since it is able to resist the different types of efforts associated with the mastication process. It should be stressed that, even for the same type of prosthesis, the requirements may vary, depending on the retention method [6,7]. For example, according to Saverio et al. [8], cement-retained configurations are generally considered to be more fracture-resistant than screw-retained ones since they better distribute the occlusal forces evenly across the implant.

Several strategies have been investigated to improve the mechanical resistance of ceramics. For vitroceramics, the control of the size and morphology of the crystallites within the vitreous matrix is crucial, since it delays or suppresses the propagation of cracks through the absorption of the fracture energy [9]. In addition, mechanical resistance can be improved by the reinforcement of the vitroceramic with ceramic particles (e.g., zirconia and alumina) [10,11,12,13,14,15,16]. For alumina, this is achieved by a high level of control of the chemical composition, porosity, density and grain size (≤4.5 µm) [17]. Finally, in zirconia, the control of the phase transformation by the addition of stabilizing oxides (e.g., Y_2_O_3_) leads to an increased fracture toughness [18,19]. In fact, it is known that the addition of 3 mol.% of yttria to tetragonal polycrystalline zirconia (3Y-TZP) results in the retention of this phase at room temperature [20]. The application of an external load induces a stress concentration at the crack’s tips, leading to the local transformation of the metastable tetragonal phase to a stable monoclinic phase with a consequent volume expansion (approximately 4.5%) [21]. This transformation toughening mechanism sets the cracks into compression, retarding their growth which subsequently improves strength [20].

Besides the high fracture toughness, 3Y-TZP presents excellent properties such as high flexural strength, excellent ionic conductivity, thermal and chemical stability, good biocompatibility, and corrosion resistance. Additionally, it does not induce allergic reactions. 3Y-TZP restorations are usually coated with a glaze (glass veneer) in order to achieve optical properties similar to those of the adjacent teeth (e.g., color, translucency) [4,22]. However, these restorations often suffer adhesive chipping of the coating, which results from the difference in materials’ coefficient of thermal expansion (CTE) [22]. In addition, several in vitro studies have demonstrated that these coatings usually lead to abnormal wear of the antagonist teeth due to the interlocking of third-body particles between the sliding surfaces [1,4].

In the dentistry industry, ceramic-based materials are usually processed by subtractive manufacturing (SM) techniques, namely through milling, diamond grinding, laser ablation or ultrasonic machining. All of them involve the removal of material from ceramic blocks, differing in terms of the cutting tools used (drill, diamond disk, laser beam and high-frequency vibrations/abrasives, respectively). The mostly used method for the production of zirconia pieces is milling, which is based on computer-aided design (CAD) and computer-aided manufacturing (CAM) technologies. Pre-sintered or fully sintered blocks are processed through a computer numeric controlled machine to obtain pieces of a specific size and shape. The use of this methodology entails several drawbacks: high material waste; limited accuracy in the production of parts with intricate internal details which impairs the reproduction of fine details; high processing time; and the cost of fabrication of complex pieces [23]. Additive manufacturing (AM), also known as 3D printing, has emerged as a promising technique to produce long-term dental structures. AM uses a computerized 3D model to produce pieces following a layer-by-layer approach (each layer is deposited one on top of the previous, consecutively, until achieving a 3D part). Its innumerous advantages when applied to this field have been highly reported in the literature [24]. Dental restorations are usually expensive, not only due to the price of the raw material, but also because they are customized products and their production involves a high workload. This restricts their accessibility to the general population, especially to the most disadvantaged sectors of society. The use of AM in the dental products’ digital production system (industry 4.0) presents innumerous economic, environmental, and social profits, such as low energy consumption, low production time and material waste, high efficiency, and the possibility of implementing a decentralized production, allowing mass production. Due to a rising life expectancy, higher awareness about oral health problems and the increasing importance of aesthetic issues, there is a high demand for dental restoration products. It is predictable that the generalization of AM use in dentistry will have a quite positive impact on the healthcare sector, with consequent benefits to the population. Therefore, it is important to understand how the current dental materials and novel materials can be processed by this technology so that they can be integrated into the production systems.

**Figure 2:**
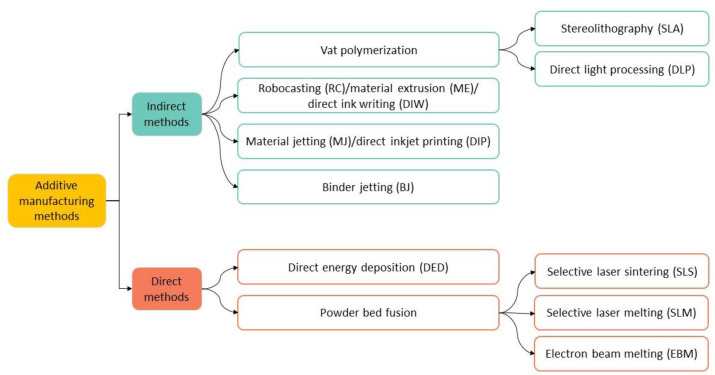
Indirect and direct AM methods. In some cases, equivalent nomenclatures are referred.

There are several AM techniques that can be used to produce 3D-printed dental structures. As shown in Figure 2, they can be divided into two main groups: the indirect and direct methods. In the first case, after printing, the part undergoes debinding and sintering, while in the latter technique no further processing after printing is required.

Indirect methods are preferable since they lead to a higher degree of consolidation of the parts and reduce the risk of cracking. In fact, contrarily to direct methods, where the material is melted by a focused energy source (e.g., plasma arc, laser, or electron beam) and simultaneously deposited in non-controlled temperature conditions, and which tend to suffer from fast cooling, indirect methods involve gradual heating protocols, reducing thermal shock. It should be stressed that the final results, namely the accuracy/resolution and the mechanical and aesthetic properties of the parts produced by a given technique, are influenced by a number of factors such as the raw materials and binders used, printing parameters (e.g., layer height, printing velocity and orientation, nozzle/light source characteristics), and post-printing treatments such as debinding and sintering [25,26]. Debinding consists of the elimination of the organic compounds that are mixed with the ceramic powders in order to allow printing. This is typically performed by heating the printed part in an oven at a temperature above the glass transition temperature of those compounds. The temperature required for removing the resin can vary depending on the specific type of resin, but usually ranges between 80 and 120 °C. Subsequent sintering is performed at higher temperatures, typically at 1500–1600 °C [20], but these temperatures can be lower if zirconia is mixed with vitroceramic materials [16].

There are few studies in the literature addressing the use of direct methods to process zirconia-based materials. Several authors state that, since zirconia presents low thermal conductivity, high melting temperature, and low thermal shock resistance, it is difficult to obtain pieces without defects (e.g., cracks and large open pores) [27].

This review will focus on the most widely used methods for the 3D printing of zirconia-based materials for dental applications, which fall in the indirect methods’ group. Below, we explain the working principle of vat polymerization, which includes stereolithography (SLA) and direct light processing (DLP), robocasting (RC), material jetting (MJ) and binder jetting (BJ) (see Figure 2) [27,28,29,30].

### 1.1. Vat Photopolymerization

In vat polymerization technology, a photopolymerizable liquid is placed inside a vat and cured by light action (UV light or UV laser, typically 380–405 nm) in accordance with a pre-defined design (CAD file) of the piece. The oligomers/monomers (epoxy or acrylic and methacrylic) present in the liquid are crosslinked in the presence of photo-initiators in thin layers, on/under a submersed platform, depending on if it is a top-down/bottom-up approach. After building each layer, the platform is re-submerged in the leftover resin to allow its spreading over the vat. The process is repeated until all the layers that constitute the piece are stacked and cured. There are two types of vat photopolymerization technologies: stereolithography (SLA) and direct light processing (DLP), which basically differ in the type of light used and in the way the light reaches the 3D-printed object [23].

▪ Stereolithography (SLA)

**Figure 3:**
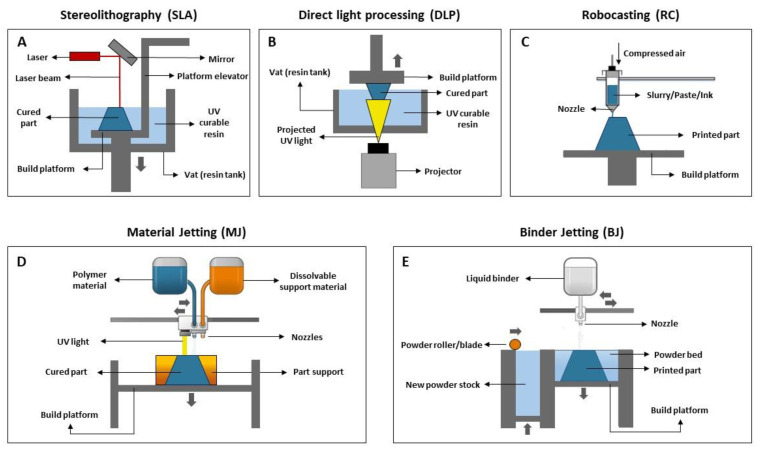
Scheme of AM indirect methods used for dental applications (adapted from [31,32]). (**A**) Stereolithography (SLA), (**B**) Digital light processing (DLP), (**C**) Robocasting (RC), (**D**) Material Jetting (MJ), (**E**) Binder Jetting (BJ).

Stereolithography (SLA) uses a laser beam that moves across the forming layer surface, leading to a localized polymerization of the photosensitive resin. It allows for the production of complexly shaped pieces with high dimensional accuracy and surface quality since the curing of the resin is performed spot-by-spot. Generally, the laser is not directly focused onto the resin, being deflected by a non-fixed mirror galvanometer that directs the beam to a specific point (Figure 3A). There are two main types of SLA printers: one where the laser is located above the vat and points down into the resin, and another where the laser is placed below the vat and points upwards into the resin. SLA is the technique most widely used to produce pieces for dental applications since it provides the highest accuracy and resolution, as well as flawless surface finishing.

▪ Direct light processing (DLP)

Digital light processing (DLP) (referred to by some authors as mask projection stereolithography (MPSL), maskless projection slurry stereolithography (MPSS) or 3D slurry printing (3DSP)) use a stationary digital micromirror device that reflects and focuses UV light, curing a complete layer of resin at once. Contrarily to SLA, layer curing in DLP is not performed spot-by-spot but rather through a single projection in a plane where photopolymerization occurs, resulting in a faster printing rate (Figure 3B) [33]. Since the projector is a digital screen, each layer is made up of a number of pixels and may be described as a set of little rectangular bricks known as voxels. The intensity of the UV light can be adjusted, allowing us to control its effect on the resin. DLP offers good feature resolution (down to several micrometers) and accuracy, being suitable to build larger and more intricate parts at higher speeds than SLA. Regarding the building configuration, DLP can produce pieces in a bottom-up or top-down setup. In the first case, the piece is cured under an inverted platform and dipped into a thin slurry layer deposited in the vat rather than entirely immersed in the liquid resin, as happens in the second case. Bottom-up setup is less expensive since it requires a lower amount of slurry to produce the desired piece. On the other hand, printers with a top-down setup allow the production of larger parts.

### 1.2. Robocasting (RC)

Robocasting (RC), also known as material extrusion (ME) or direct ink writing (DIW), involves the use of a stable slurry/paste/ink with high solid loading that is extruded through a nozzle (Figure 3C). This moves over a platform to directly “write” the desired shape in a layer-by-layer manner until the piece is complete. It should be noted that after the extrusion of one layer, it is not necessary to wait for the material’s solidification or drying before depositing the next layer. RC may be challenging due to issues related to the printability of pastes. The paste optimization process is crucial since an ink with adequate composition, rheological properties (relatively low viscosity under stress) and excellent shape retention capacity (high elastic/storage modulus, high yield stress) should be obtained to ensure its adequate extrusion and that, during deposition, each layer supports its own weight without collapsing. Pastes with high solid loading allow the production of bulk samples with high densities.

### 1.3. Material Jetting (MJ)

Material jetting (MJ), also known as direct inkjet printing (DIP), is a process that operates in a similar way to the 2D ink-jetting process. However, instead of a filament, the material is deposited in the form of droplets. In this technique, a printhead moves horizontally (*x*–*y* axis), and droplets of a photosensitive material are deposited onto the building platform, at that point being directly cured under UV light. The piece is built in a layer-by-layer approach. In brief, first the liquid resin is heated 30–60 °C to achieve a suitable viscosity for printing. Then, the print head travels over the building platform and hundreds of tiny droplets of photopolymer are jetted/deposited inside a support (see Figure 3D). A UV light source that is attached to the print head cures the deposited material, solidifying it and creating the first layer of the part. After each layer is complete, the building platform moves downwards one in layer height, and the process is repeated until the whole part is completed. After printing, the support material (which is soluble in specific solvents) is removed. MJ is a fast and economical AM technique with minimal waste and high flexibility which allows for a high level of accuracy in the deposition process.

### 1.4. Binder Jetting (BJ)

In binder jetting (BJ) (Figure 3E), a recoating blade spreads a thin layer of powder over a building platform. Then, the printhead selectively deposits droplets of a liquid binding agent that bonds the powder particles together, according to the CAD project. After the application of the binder, the building platform moves down, and another layer is built over the previous, following the same procedure.

This process is fast, simple, and cheap, requiring the use of materials in the powder form. Additionally, it prevents the formation of residual stresses in the produced parts since there are no thermal inputs (no light source is used). However, it leads to pieces with low mechanical resistance and is not suitable for use to produce structural parts.

## 2. Materials and Methods

To conduct this review, the authors formulated the following question: “What is the state-of-the-art regarding the use of 3D printing technologies to produce zirconia-based materials for dental applications?”

### 2.1. Protocol

This systematic literature review followed the PRISMA (Preferred Reporting Items for Systematic Reviews and Meta-Analyses) guidelines [34].

### 2.2. Eligibility Criteria

The works considered in this study were gathered based on the following inclusion criteria:

- Studies that include zirconia based materials;
- Studies that use additive manufacturing techniques/3D printing technologies;
- Studies that evaluate the properties of the printed materials;
- Studies that focus on materials used for dental applications;
- Articles published in English;
- Articles published in peer-reviewed journals;
- In vitro studies.

Literature reviews, systematic reviews, case series, manufacturer reports, and conference abstracts were not considered.

### 2.3. Information Sources and Search Strategy

The search for this review article was conducted on three different online databases: PubMed, Scopus and Web of Science. The last search was carried out on 14 December 2022. Regarding the year of publication, no restriction was applied. The keywords used for searching were: (Additive Manufacturing OR 3D printing) AND (robocasting OR direct ink writing OR material extrusion OR stereolithography OR digital light processing OR material jetting OR direct inkjet printing OR binder jetting) AND (dental materials OR dental applications OR dentistry) AND (zirconia OR zirconia composites OR ceramic composites) AND (leucite OR lithium disilicate OR vitroceramics).

**Figure 4:**
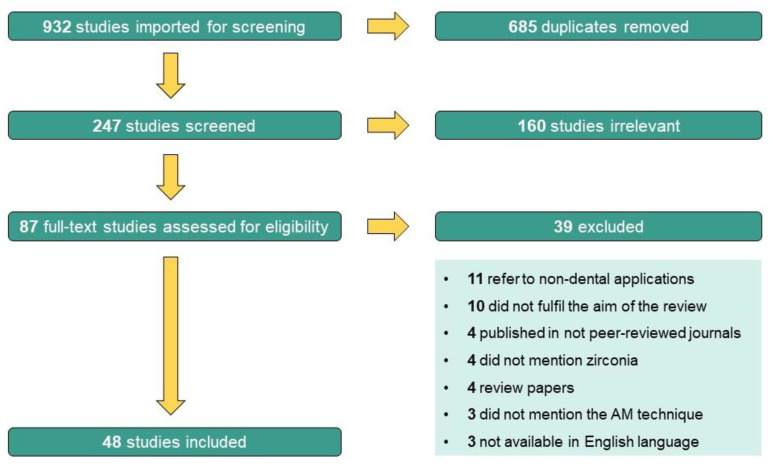
PRISMA flow chart diagram.

This search was conducted using the Mendeley software (version 1.19.8). Two independent researchers (A.C.B. and A.P.S.) started by analyzing titles and abstracts identified in the initial search for their relevance and fulfilment of eligibility criteria. Firstly, articles were classified as “include”, “exclude”, or “uncertain”. Then, the full-text articles of the “include” and “uncertain” records were analyzed for further eligibility screening by the same researchers, who worked independently. When there were discrepancies in screening of titles/abstracts and full-text papers, the two researchers discussed the issue. In case of disagreement, the opinion of a third researcher (C.F.P.) was obtained, and a decision was reached. Finally, after full-text review, some articles were excluded and the reasons for rejecting the studies were highlighted (see Figure 4).

### 2.4. Data Extraction and Results Achievement

This review article is focused on 3D printing technologies used to produce zirconia-based materials for dental applications. In order to summarize the retrieved information, a table with the following information was built: author (publication year), manufacturing technology, ceramic material, dental application, studied properties, and main results. Then, a descriptive analysis was performed to compare and point out similarities and differences among the studies.

## 3. Results

The initial search yielded 932 potentially relevant articles, of which 685 were excluded as duplicates. Following a title/abstract review and subsequent full-text review, 160 additional articles were excluded as they were considered irrelevant to the main aim of this review. Of the 87 remaining articles assessed for eligibility, after full-text screening, 39 more studies were excluded due to the following reasons: 11 were studies whose application was not for dental applications, 10 did not fulfil the aim of the review, 4 were articles published in not peer-reviewed journals, 4 did not mention zirconia as one of the studied materials, 4 were review papers, 3 did not mention the additive manufacturing technique used and 3 were not available in the English language. This screening process led to the inclusion of 48 studies in the review. The PRISMA flow chart diagram below (Figure 4) depicts the selection process.

**Figure 5:**
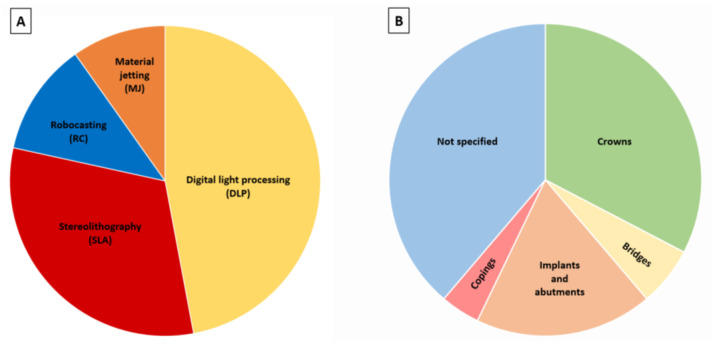
(**A**) Most studied AM techniques; (**B**) Dental applications.

The considered papers were published between 2010 and 2022, despite no restrictions being imposed regarding the publication dates. This reveals the innovative character of the subject, whose interest has increased even more in the last four years: 8 articles were from 2019, 9 from 2020, 15 from 2021 and 9 from 2022. Most of the studies are published in materials or dental materials journals and are focused on yttria-stabilized tetragonal zirconia. Most of the studies were performed in Asia (total 28), mostly from China (21), but also from South Korea (5) and Saudi Arabia (2). In Europe (total 13), studies from Portugal (4), Belgium (3) and the Netherlands, Germany and Italy (2 in each case) were found. From North America only 7 studies were found (United States (6) and Canada (1)). The most productive group was that of Revilla-León et al. which has published 5 papers [29,35,36,37,38] since 2020 that, at present, have received 57 citations. Concerning the additive manufacturing technique, 24 used digital light processing (DLP), 16 used stereolithography (SLA), 6 used robocasting (RC)/material extrusion (ME)/direct ink writing (DIW), 5 used material jetting (MJ)/direct inkjet printing (DIP) and 1 used 3D gel deposition (Figure 5A). In terms of the dental application, some works centered on the production of materials for crowns (16), bridges (3), implants and abutments (9) and copings (2) (Figure 5B). However, some studies did not report any specific dental application (19). Several properties were studied among the selected studies: cure depth, density, shrinkage, dimensional accuracy, trueness and precision, internal fit and marginal adaptation, translucency, surface roughness, microstructure, mechanical properties (hardness, fracture toughness, flexural strength, elastic modulus, fracture load), and wear (antagonist teeth and prosthetic materials).

Table 1, Table 2, Table 3, Table 4 and Table 5 précis the main findings of studies involving the 3D printing of zirconia-based materials.

## 4. Discussion

Most of the studies reported in the literature concerning the production of zirconia-based dental pieces by 3D printing are focused on vat polymerization techniques, namely digital light processing (DLP) and stereolithography (SLA) [23,81,82], which allow the curing of consecutive layers of a photosensitive polymeric material mixed with ceramic particles. Both techniques usually lead to the production of parts with high accuracy and resolution, smooth surface finishing and fine building details [83,84,85]. Although there are far fewer studies addressing robocasting (RC)/direct ink writing (DIW) and material jetting (MJ)/direct inkjet printing (DIP), these techniques have also been reported as potentially suitable processing methods to be used in dentistry. Below, the main properties of the materials processed by the different techniques will be addressed, as well as some aspects affecting them.

### 4.1. Cure Depth

DLP and SLA are similar technologies that use light (UV or laser), and therefore the nature and size of the ceramic particles and the pastes’ solid loading are key factors in the curing process of the layers. Since zirconia presents a high refractive index (2.1, which is 20–27% higher than silica and alumina [86]), it usually induces significant scattering of the incident light during polymerization of the resin that contains it, decreasing the cure depth [42,43,87,88]. As for the size, smaller particles also tend to increase light scattering [89], threatening the final result. A high solid load (≥60 vol.%) usually leads to low cure depth, resulting in extra ceramic material loosely adhering to the final printed piece [42,90]. Besides, it translates into a high viscosity, which may impair the resin flow during each layer formation and the cleaning of the resin tank. Ideally, pastes should present a viscosity lower than 5 Pa·s [91], comparable to unloaded resins. However, solid loading must be enough to ensure the obtention of pieces with high density after sintering.

The ceramic suspension used for DLP/SLA must be stable, i.e., the ceramic particles must be homogeneously and effectively dispersed in the photocurable resin for a reasonable period (e.g., hours to days) and not suffering from sedimentation. To achieve suspensions’ stability/homogeneity, several approaches can be followed such as the addition of dispersants and other additives to the slurry, particles’ coating, ultrasonication, vacuum drying, ball milling, and acid treatment [88].

### 4.2. Density and Mechanical Properties

**Figure 6:**
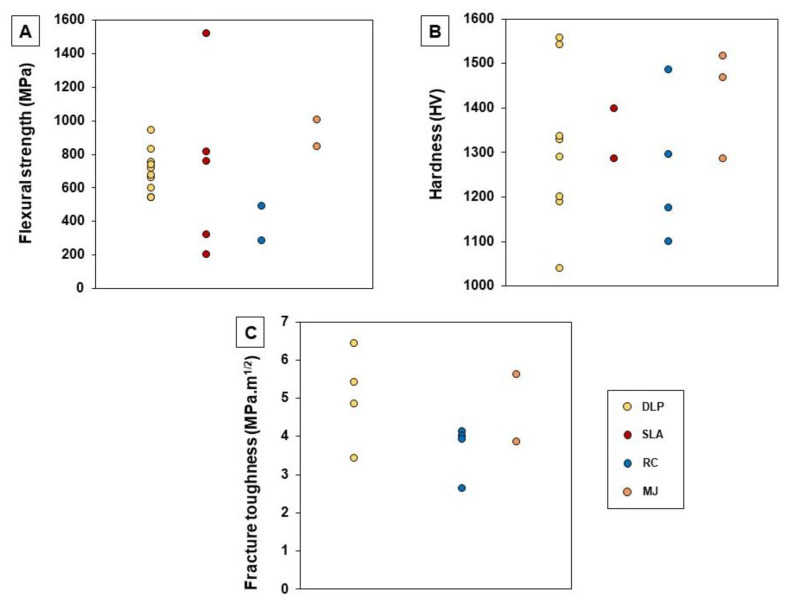
Dispersion charts of retrieved data regarding flexural strength, hardness and fracture toughness from the selected studies presented on Table 1, Table 2, Table 3 and Table 4. (**A**) Flexural strength; (**B**) Hardness; (**C**) Fracture toughness.

For DLP and SLA, light curing slurries have been prepared with ceramic powder loadings that range from 34.5 to 58.0 vol% [42,43,44,45,46,48,49,51,52,53,54,56,57,59,60,63,65,66,67,77]. Values between 98 and 99.8% were obtained for the materials’ theoretical density (TD) [40,41,44,45,48,49,52,53,57,60,67]. As expected, higher solid loadings led to a higher density of the sintered pieces [42,59,90]. Similarly, in general, the mechanical properties improved for higher solid amounts [42,57,59]. One of the most commonly evaluated mechanical properties was flexural strength (see Figure 6A). This was found to be, in most of the cases, lower than that of zirconia pieces produced by conventional manufacturing techniques (200–831 MPa [35,39,40,41,42,45,46,48,49,51,53,57,60,63,77] vs. 900–1200 MPa [20,46,92,93]). However, pieces with a higher flexural strength (943–1519 MPa) [36,41,49,77] were also obtained by some authors using DLP or SLA. These higher values may be a result of the improvement of slurry composition, which leads to more adequate viscosity, as well as the optimization of the parameters for debinding and sintering processes. Hardness and fracture toughness were also widely characterized: their values are reported to encompass 1038–1556 HV and 3.43–6.42 MPa.m^1/2^, respectively (see Figure 6B,C) [35,39,40,43,44,46,47,49,52,53,57,60]. These values are similar to those found for zirconia parts obtained by conventional manufacturing methods [1,2]. Regarding zirconia composites, there are still only few studies where its production by 3D printing is addressed. Wu et al. [44] and Coppola et al. [51] used DLP in the manufacturing of Al_2_O_3_-ZrO_2_ (ATZ) composites and observed that the mechanical properties improved significantly compared to full zirconia materials, achieving hardness values in the range of 1290–2141 HV. More, Coppola et al. [51] verified that the values decrease with the increasing zirconia content.

Other techniques, like robocasting (RC) and material jetting (MJ), also present high potential in the dentistry field. However, information available in the literature related to their use in processing zirconia for those applications is limited. As referred to in Table 3 and Table 4, the TD of the materials produced by these techniques fall in the range of 94.0–98.1% for RC samples [1,69,70,72] and 96.0–99.7% for MJ samples [73,74,75,76]. Their mechanical properties, namely flexural strength, hardness and fracture toughness are, in most cases, comparable to those found when vat polymerization methods (DLP and SLA) are used (see Figure 6A–C) [1,69,72,73,74,75,76,94].

### 4.3. Defects

Concerning the defects of 3D-printed samples, some studies reported the presence of pores and cracks/fractures on the samples’ surface. Li et al. [61] obtained ZrO_2_ pieces for bridges and implants by SLA and found cracks on the outer surface that suffered propagation. Besides, they observed the presence of pores (200–400 nm) distributed all over the surface. In the work of Osman et al. [41], cracks, microporosities and interconnected pores with sizes ranging from 196 nm to 3.3 µm were also observed. Revilla-León et al. [36] produced ZrO_2_ pieces by SLA but SEM images revealed that there was no evidence of cracks, fracture surfaces, or flaws. Instead, an irregular surface with pits of 10–40 µm was detected. Marsico et al. [47] showed that DLP-sintered pieces presented fractures that began at layer lines and surface defects (pores). Jang et al. [42] concluded that surface cracks decreased with the increasing content of ZrO_2_, leading to the formation pieces with higher mechanical resistance. Xiang et al. [65] produced pieces using SLA and observed a weak bonding strength among the successive layers as well as surface defects resultant from the process of separating the piece from the building platform. Apart from impairing the aesthetic properties of the final piece (e.g., decreasing the translucency), these surface defects (internal flaws such as pores and agglomerations) also increase the failure probability of the restorations. These authors performed three-point bending tests and observed two types of fracture modes after testing: fractures due to stress concentration and splintering due to crack deflection. In another work where pieces for crowns and fixed prosthesis were produced by SLA [38], it was found that, as expected, the higher the porosity was, the lower the fracture load, flexural strength and flexural modulus would be: samples with 0% porosity showed fracture loads of 1132.7 N, flexural strength of 755.1 MPa and flexural modulus of 41.273 GPa, while samples with 40% porosity showed a fracture load of 72.13 N, flexural strength of 48.09 MPa and flexural modulus of 7.177 GPa. Willems et al. [76], produced pieces by MJ from a suspension with low solid loading (12.5 vol%), observing delamination, cracks, agglomerates and spherical pores. Finally, Zang et al. [80] produced restorations by 3D gel deposition and cold isostatic pressing and determined the fracture force before and after fatigue tests (5,000,000 cycles ≈ 20 years of clinical service). They observed that the first samples led to a higher fracture force (≈8000 N) than the latter (≈7000 N) (both before and after fatigue testing without statistically significant difference) due to the fine-grained microstructure without visible microscopic voids.

### 4.4. Aesthetic Features

Since 3D printing manufacturing techniques involve layer-by-layer deposition, and aesthetic requirements are quite relevant in dental applications, it is critical to ensure that the interfaces between layers, in the final product, are barely defined. Revilla-León et al. [36] printed parts by SLA and found a layer strand texture with a smooth depression between the layers (no more than 5 and 10 µm). Silva et al. [68] produced fixed partial dental structures by RC and observed a surface with a “stair stepped” appearance and the presence of cracks derived from the drying step. Contrarily, Özkol et al. [73] produced dental bridges by MJ and observed a smooth surface without “stair steps”. In turn, Li et al. [43] and Kim et al. [48] produced dental crowns via DLP and verified that the interlayered structure disappeared after sintering. It should be stressed that the application of glass veneers over the zirconia restorations, which is a common procedure to improve aesthetics, generally hides the layered structure, minimizing the importance of this issue.

### 4.5. Dimensional Accuracy and Resolution

**Figure 7:**
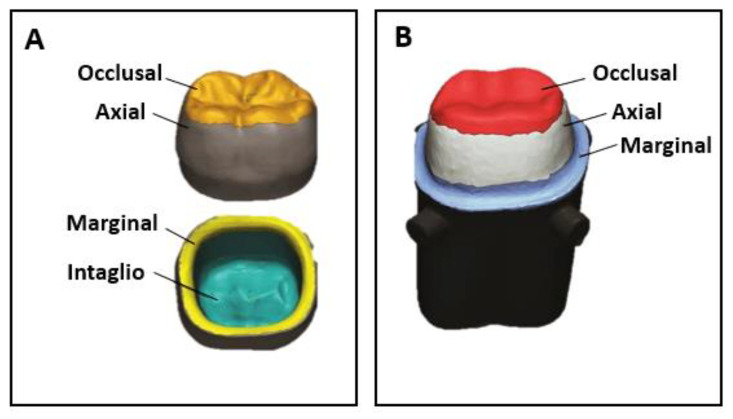
(**A**) Different areas of prosthetic crowns relevant for the evaluation of trueness and precision; (**B**) Internal fit and marginal adaptation (adapted from [25]).

Other important aspects that must be evaluated after the printing process are the accuracy and resolution of the produced pieces. These are influenced by a wide range of factors, e.g., technology used, post-treatment procedures, particle size and layer thickness [95]. Accuracy evaluates two parameters: trueness and precision. While trueness is defined by the deviation of the produced printed piece from its desired dimensions, precision measures the consistency between repeated prints (i.e., the ability to produce the same part with the same dimensions in consecutive prints) [83]. A high precision in the production of dental restorations ensures an appropriate fit and an adequate biological response. Resolution concerns the detail level achieved in three dimensions and depends on the number of points that a 3D printer can effectively reproduce. Although the accuracy and resolution of the process is crucial for the success of a dental restoration, the number of studies on this topic is still quite limited in the literature. Lerner et al. [50] produced ZrO_2_ crowns by DLP and SM and evaluated their trueness and precision. They found that crowns produced by SM showed higher trueness than DLP crowns. Regarding precision, both techniques led to similar results in terms of the quality of interproximal contact points and marginal closure. Lüchtenborg et al. [79] compared the accuracy of fixed dental prostheses produced by SLA, DLP, MJ and SM and concluded that SM led to the most accurate pieces. More, a prototype piece of DLP equipment resulted in surface deviations higher than 100 µm, representing the least accurate method. However, this depended on the DLP printer/characteristics of the material used, since the authors reached better results with a commercial DLP printer that uses a specific material optimized for that printer. Also, Moon et al. [55] observed that DLP pieces produced for dental copings suffered higher thermal shrinkage and presented lower accuracy than those produced by SM. In another study performed by Wang et al. [62], ZrO_2_ crowns were produced by SLA and SM. They verified that the trueness of the external surface, intaglio (interior) surface, marginal area, and occlusal surface were similar in both cases (Figure 7A). Additionally, Kim et al. [78] used DLP, SLA and SM to produce ZrO_2_ crowns and did not observe statistically significant differences at the inner surface area, concluding that the trueness of intaglio crown surface was similar regardless of the manufacturing method. However, differences regarding the trueness of the occlusal, margin and axial areas were found: at the occlusal area, DLP samples showed the lowest mean values, being statistically significantly different from SLA and SM-4YZ samples; at the marginal area, both DLP and SLA samples presented significantly higher values than SM groups; finally, at the axial area, a significantly lower value was found for SLA samples when compared to SM samples (4YZ and 5YZ). Accuracy is crucial to ensure a suitable internal fit and the marginal adaptation of provisional crowns and fixed-dental prosthesis (Figure 7B). A suitable internal fit (≤300 µm) improves the retention, resistance, and durability of the restorations, while an adequate marginal adaptation (≤120 µm) impairs microleakage, the dissolution of fixation cement, bacterial plaque accumulation, secondary caries and periodontal inflammation [56]. The last is the principal measurement metric used for dentistry since it has a significant impact on the longevity of dental restorations [46]. Meng et al. [56] produced ZrO_2_ crowns by DLP and observed that the internal fit and marginal adaptation of fixed crowns were 239.3 ± 7.9 µm and 128.1 ± 7.1 µm, respectively, being close to clinical standards. However, crowns produced by SM led to values of 68.5 ± 3.9 µm for internal fit and 71.6 ± 2.8 µm for marginal adaptation, being more reliable than those produced by DLP. In another study, Hsu et al. [46] measured the marginal adaptation of premolar teeth produced by DLP and SM and observed that DLP led to higher values (98.9 µm) than SM (72 µm). However, they were lower than 120 µm, which is the maximum value acceptable for clinical use. Li et al. [63] produced crowns by SLA. Although they presented suitable mechanical properties, they were considered non-ideal for dental application since it was observed a cement space of 169.58 µm in the marginal area. Finally, Abualsaud et al. [25] studied the internal fit, marginal adaptation, precision and trueness of zirconia crowns produced by SLA and SM and reported similar internal fit and marginal adaptation between pieces produced by both methods. Regarding trueness, SLA crowns revealed better occlusal (8.77 ± 0.89 µm) and axial (14.77 ± 2.03 µm) trueness than SM crowns (14.78 ± 2.23 µm and 20.37 ± 4.49 µm, respectively), while SM crowns showed better intaglio trueness (20.29 ± 3.82 µm) than SLA crowns (23.90 ± 1.60 µm). Furthermore, SLA led to more precise crowns (9.59 ± 0.75 µm) than SM crowns (17.31 ± 3.39 µm).

### 4.6. Bonding Strength between Materials

When ZrO_2_ crowns are damaged and/or need to be refurbished, the application of glass ceramic copings can avoid their replacement. This procedure allows us to improve aesthetic properties like translucency and color, is less harmful to patients, leading to lower recovery times, and involves lower costs. Its success is determined, among other factors, by the bonding strength between the two materials. Moon et al. [55] produced pieces of porcelain-fused zirconia, the being later obtained by DLP or SM. They observed adhesive failure (debonding) between the two interfaces in both cases. However, the bond strength to zirconia produced by DLP was significantly higher than the strength of the bond to zirconia processed by SM (35.12 ± 4.09 MPa vs. 30.26 ± 5.20 MPa). 

### 4.7. Tribological Behavior

Finally, although wear is a subject of great relevance for dental materials, few studies address the tribological behavior of prosthetic materials obtained by 3D printing and antagonist teeth. Kim et al. [78] produced 3YZ pieces for full-contour monolithic crowns via DLP and SLA and performed chewing simulation tests against human molars. They observed that the antagonist’s wear volume loss was 2.06 ± 1.24 mm^3^ (DLP) and 1.74 ± 1.20 mm^3^ (SLA), similar to the values observed with samples produced by SM (2.51 ± 2.13 mm^3^ and 2.40 ± 1.66 mm^3^ for SM-4YZ and SM-5YZ, respectively). Branco et al. [1] conducted a study where ZrO_2_ pieces were produced by RC methods and compared with those produced by SM. In chewing simulation studies carried out in artificial saliva against natural human teeth cusps, it was found that any of the prosthetic materials suffered wear. Contrarily, all ZrO_2_ pieces induced wear on the cusps, this being significantly higher in the case of SM samples. These authors also applied a glaze finishing over RC and SM pieces and observed that, contrarily to the uncoated surfaces, both glazed surfaces and dental cusps suffered wear. The wear of the cusps was higher than that found on unglazed specimens. Moreover, RC glazed pieces induced less wear on the antagonist cusps than SM glazed pieces.

### 4.8. Printing Orientation

One important factor that must be considered when producing pieces by 3D printing is the printing orientation since it will strongly influence the printed piece quality, especially in terms of accuracy, surface roughness, translucency, and mechanical properties. Depending on the printing parameters, the adhesion between layers may be different from the adhesion between lines in the same layer. Xiang et al. [65] concluded that samples printed by SLA in an upright way led to higher density and translucency than when printed horizontally, while samples printed horizontally led to excellent accuracy and mechanical properties. In another study, Coppola et al. [57] produced samples via the DLP method and found that plane they showed higher flexural strength when tested perpendicularly to the printing (≈751 MPa) than when tested parallelly (≈675 MPa) to the printing plane. Similarly, Zhao et al. [53] observed that the flexural strength of samples printed by DLP in the horizontal direction was higher than those printed in the vertical direction (597 MPa vs. 89 MPa). Marsico et al. [47], in a study where zirconia was printed by DLP in three different orientations (0°, 45° and 90°), found that 45° orientation presented the highest indentation fracture resistance. In turn, 0° orientation showed the highest flexural strength (657 MPa) since it minimized the impact of layer line defects, these values being comparable to those reported in the literature for monolithic zirconia produced by conventional methods. Additionally, Osman et al. [41] printed ZrO_2_ implants by DLP and found that pieces printed in 0° orientation led to the highest flexure strength values (943 MPa), while the lowest values were observed for 45° printing orientation (822 MPa).

### 4.9. Ageing

The effect of ageing treatments on 3D-printed zirconia-based materials has also been scarcely addressed in the literature. Tan et al. [54] produced 3Y-TZP for implant abutments by DLP and SM and evaluated the effect of ageing (134 °C with 100% humidity, 0.2 MPa) on their physical and biological properties. They found that DLP samples presented higher initial cubic phase content and a greater rate of phase transformation than SM samples. Concerning their biological performance, the ageing treatment almost did not affect cellular behavior in any zirconia type. Only minor changes in adhered cell numbers, recorded in the function of the aging time/culturing time, were found. In another study, Léon et al. [35] investigated the effect of artificial ageing (8000 cycles between 5 °C and 55 °C) on the mechanical properties of 3Y-TZP produced by SLA and SM and observed that the flexural strength decreased ≈12% after such treatment for SLA samples and ≈37% for SM samples. Zhai et al. [77] produced ZrO_2_ samples by SLA, DLP and SM methods. They submitted the samples to ageing (134 °C, 0.2 MPa for 5 h, 10 h and 15 h) and observed that ageing times until 15 h only affected SLA samples’ properties: the flexural strength increased from 776.7 MPa in non-treated samples to 1010.3 MPa after 5 h of ageing. A decrease in the flexural strength was observed after 10 h and 15 h (913.1 MPa and 814.28 MPa, respectively). Regarding DLP and SM samples, before and after ageing, this value was around 800 MPa and higher than 1200 MPa, respectively. These authors also found that DLP samples showed zirconia grain fragments, while SLA samples presented grain pullout. Moreover, the monoclinic phase content increased with the aging time, both for DLP and SLA samples. Lastly, Wu et al. [44] produced alumina-toughened zirconia (ATZ) implants by DLP and hydrothermally treated them with steam at 134 °C for 5 h, 20 h and 40 h. After, they evaluated the aging rate and tetragonal-monoclinic phase transformation and, as expected, observed that it was lower than that of 3Y-TZP samples.

 

Overall, this review allowed us to summarize the most recent advances on 3D printing of zirconia-based dental materials, highlighting the main issues associated with the production that impact the performance of the materials. This task revealed to be challenging since it was found a great variability regarding, e.g.,:

− The characteristics of the raw materials used (including the ceramic powders and the resins), such as their concentrations, the solids content, the size distribution and shape of the particles, that determine the rheological properties of the suspension;
− The printing parameters (e.g., velocity, layer height/line width, orientation, nozzle/light source characteristics);
− The post-printing treatments (e.g., debinding and sintering thermocycles) and the surface finishing;
− The experimental procedure used for specimen characterization.

It should be stressed that the number of studies that were considered, based on the defined inclusion criteria, was relatively low, reflecting the need for more studies in this area.

## 5. Conclusions

This review aimed to present the current state of the art of additive manufacturing (AM) of zirconia-based materials for dental applications, highlighting the main outcomes and advances reached in the last few years, as well as challenges. To the authors’ best knowledge, a comparative analysis of these materials’ properties was never carried out previously. Studies on this topic are relatively scarce and quite recent (all published after 2010). It is a consensus that AM has great potential to produce dental devices, mainly due to the possibility of customization, with a lower cost compared to the conventional techniques of subtractive manufacturing (SM), such as milling, uniaxial compression and cold isostatic pressure. However, the final products still lag behind those obtained by SM methods.

Vat polymerization, namely stereolithography (SLA) and digital light processing (DLP), leads to pieces with high accuracy and resolution, revealing itself to be promising for the production of dental crowns, bridges, copings, implants and abutments. Although material jetting (MJ) and robocasting (RC) also have led to interesting results, the parts produced through these technologies usually present lower resolution/accuracy and worse mechanical resistance compared to SLA and DLP, limiting their applicability to the restoration of anterior teeth.

Despite significant progress, AM of ceramic materials is still taking its first steps since it falls short in some points, e.g., the control of the process, the preparation of suitable feedstock materials, the development of dedicated ceramic printers and the obtention of materials with adequate properties that match the dentistry requirements (e.g., aesthetics, dimensional accuracy, thermal shock resistance, chemical stability, mechanical and tribological resistance). A great effort has been mounted to adapt materials, methods, and workflows to improve the mechanical performance (by reducing surface defects such as cracks and porosity), dimensional accuracy (internal fit and marginal adaptation) and aesthetics of the restorations.

In conclusion, the research on zirconia-based materials produced by AM techniques is expanding and represents serious technological progress. However, there is still a long way to go in order to include AM techniques in the dentistry industry in a way that ensures the production of safe and durable prosthesis. Further work must be done, in particular regarding the 3D printing of vitroceramic materials reinforced with zirconia, about which there are no insights. Additionally, 3D printing of dental restorations with a multilayer approach that mimics the complex properties of the natural tooth is an important topic that has not been addressed in the literature yet.

## Figures and Tables

**Table 1:** Summary of the main results obtained for ZrO_2_-based samples produced by digital light processing (DLP).

Reference	Manufacturing Technology	Ceramic Material	Dental Application	Studied Properties	Main Results
[39]	Maskless Projection Slurry Stereolithography (MPSS) (correspondent to Digital Light Processing (DLP))	3Y-TZP (*EZU3YA-1*, *EE-Tec*)	Copings	Shrinkage, density, microstructure, hardness, flexural strength	Shrinkage of 23.5% and relative density of 98% TD. No signs of delamination and cracks. Hardness of 1328 HV. Flexural strength of 539 MPa.
[40]	Three-Dimensional Slurry Printing (3DSP) (correspondent to Digital Light Processing (DLP))	3Y-TZP (*EZU3YA-1*, *EE-Tec*)	Implants	Shrinkage, density, microstructure, hardness, flexural strength	Shrinkage ≈ 32% and relative density of 98.2% TD. No evidence of delamination and cracks. Hardness of 1556 HV. Flexural strength of 542 MPa.
[41]	Digital Light Processing (DLP) (*ADMAFLEX 2.0*; *ADMATEC*)	3Y-TZP (*TZ-3YS-E*, *Tosoh Inc.*)	Implants	Density, microstructure, surface roughness, flexural strength	Relative density of 99.8% TD. Cracks, micro-porosities and interconnected pores (196 nm to 3.3 µm). Surface roughness (Ra 1.59 ± 0.41 µm) within the range of those reported for titanium implant (1.0–2.0 µm). Flexure strength depended on the printing orientation: − 0°: 943 ± 153 MPa − 45°: 822 ± 173 MPa − 90°: 835 ± 73 MPa
[42]	Digital Light Processing (DLP) (*Octave Light R1*, *Octave Light*)	3Y-TZP (48–58 vol%) (*TZ-3Y*, *Tosoh*)	Not specified	Density, cure depth, microstructure, flexural strength	Maximum ZrO_2_ vol% possible for printing: 58 vol%. Relative density increased with the increase in vol% ZrO_2_ (83.02–92.79%TD). Cure depth decreased as ZrO_2_ vol% increased. Cracks on the surfaces increased as ZrO_2_ vol% decreased. Flexural strength increased as ZrO_2_ vol% increased (max of 674.74 ± 32.35 MPa for 58 vol%).
[43]	Digital Light Processing (DLP) (*ADMAFLEX 130*, *ADMATEC Europe BV*)	3Y-TZP (40 vol%) (*G3Y-020OO*, *Shandong Sinocera Functional Material*)	Crowns	Microstructure, hardness	Particles evenly distributed in the cured resin matrix without obvious agglomeration. Interlayered structure disappeared after binder burnout. Hardness of 1038 HV.
[44]	Digital Light Processing (DLP)	ATZ (38.5 vol% ZrO_2_) (*HWYA*, *Guang Dong Huawang Zirconium Materials*)	Implants	Density, hardness, fracture toughness, ageing rate, phase transformation	Relative density of 98.11%TD. Hardness of 1290 HV. Fracture toughness of 6.42 MPa.m^1/2^ ATZ samples showed lower aging rate and phase transformation depth than 3Y-TZP.
[45]	Mask Projection Stereolithography (MPSL) (correspondent to Digital Light Processing (DLP))	YSZ (40 vol%)	Crowns	Density, flexural strength	Relative density of 99.3% TD. Flexural strength of 541 MPa.
[46]	Three-Dimensional Slurry Printing (3DSP) (correspondent to Digital Light Processing (DLP)) Subtractive Manufacturing (SM) (*CORiTEC 245i*)	3DSP: YSZ (75 wt%–34.5 vol%) SM: YSZ (*Copran Zr-i Monolith A3—Zirkonblank*, *Whitepeak Dental solution*)	Crowns	Marginal adaptation, hardness, flexural strength	Marginal adaptation for 3DSP samples higher (98.9 µm) than for SM samples (72 µm), being both less than the threshold value (≤120 µm) Hardness for SM samples higher (1238.8 HV) than for 3DSP samples (1189.4 HV). 3DSP samples’ flexural strength of 716.76 MPa.
[47]	Digital Light Processing (DLP)	5Y-PSZ	Not specified	Density, hardness, fracture toughness, flexural strength, microstructure	Relative density of 99.3% TD. Samples printed in different orientations (0°, 45° and 90°): − Hardness independently of the printing orientation (1336 ± 32 HV). − 45° orientation presented the highest fracture toughness (4.85 ± 0.46 MPa.m^1/2^) − 0° orientation showed the highest flexural strength (657 ± 84 MPa). − 0° and 90° exhibited more layer line-related failures. Fracture frequently initiated at layer lines.
[48]	Digital Light Processing (DLP)	4Y-PSZ (50 vol%) *(Zpex4*, *Tosoh*)	Crowns	Density, microstructure, flexural strength	Relative density of 99.4% TD. No visible interfaces between the layers. Flexural strength of 831 MPa.
[49]	Digital Light processing (DLP) (*QuickDemos Company*) Subtractive Manufacturing (SM) (*Wieland Zenostar mini*, *Ivoclar Vivadent*)	DLP: Y-TZP (58 vol.%) (*QuickDemos Company*) SM: Y-TZP (*Zenostar*, *Ivoclar Vivadent*)	Not specified	Density, microstructure, fracture toughness, flexural strength	DLP samples’ relative density of 99% TD. Both materials with similar microstructures considering grain size, phase composition, and defects. DLP samples’ fracture toughness of 5.4 ± 0.5 MPa.m^1/2^ Flexural strengths of 737.4 ± 99.5 MPa (DLP) and 984 ± 94.7 MPa (SM).
[50]	Digital Light Processing (DLP) (*Cerafab S65*^®^, *Lithoz*) Subtractive manufacturing (SM) (*DWX-52D*^®^, *DGShape*, *Roland Company*)	DLP: 3-TZP (*LithaCon 3Y 230 D*; *Lithoz*) SM: LithaCon 3Y 210^®^ (*Lithoz*)	Crowns	Trueness, precision	SM crowns had higher trueness than DLP crowns. DLP and SM groups presented similar precision (quality of interproximal contact points and marginal closure).
[51]	Digital Light Processing (DLP) (*ADMAFLEX 130*, *ADMATEC Europe BV*)	Alumina-zirconia (AZ) composites (15, 50, 85 vol% ZrO_2_) with 40 vol%: α-alumina (*AdmaPrint A130*) 3Y-TZP (*AdmaPrint Z130*)	Not specified	Microstructure, hardness, flexural strength, elastic modulus	Homogeneous microstructure with good second-phase particles distribution. Hardness of 1530–2141 HV (values decrease with the increasing zirconia content). Flexural strength of 415–843 MPa. Elastic modulus of 188–318 GPa.
[52]	Digital Light Processing (DLP) (*Ceramatrix*, *QuickDemos Company*) Subtractive Manufacturing (SM) (*Wieland Zenostar mini*, *Ivoclar Vivadent*)	DLP: Y-TZP (58 vol%) (*QuickDemos Company*) SM: Y-TZP (*Ivoclar Vivadent*)	Not specified	Density, microstructure, hardness, fracture toughness	Relative density of 99% TD (both for DLP and SM samples). SLA and SM samples presented similar grain size and crystalline phase composition. Hardness of DLP samples lower (1189 HV) than for SM samples (1248 HV). Fracture toughness similar for DLP (3.43 ± 0.29 MPa.m^1/2^) and SM samples (3.44 ± 0.23 MPa.m^1/2^).
[53]	Digital Light Processing (DLP)	5Y-PSZ (78 wt%–38.5% vol%)	Implant abutment	Density, hardness, flexural strength	Relative density of 99.48% TD. Hardness of 1542 HV. Flexural strength of the sample printed in the horizontal direction (597 MPa) was better than that in the vertical direction (89 MPa).
[54]	Digital Light Processing (DLP) (*CeraLab-P60*, *QuickDemos Company*) Subtractive Manufacturing (SM) (*Wieland Zenostar mini*, *Ivoclar Vivadent*)	DLP: 3Y-TZP (58 vol%) (*QuickDemos Company*) SM: 3Y-TZP (*Ivoclar Vivadent AG*)	Implant abutment	Effect of accelerated aging on physical and biological properties	DLP samples showed higher initial cubic phase content and rate of phase transformation than the SM samples. Aging did not affect cellular behavior in any zirconia type, except for minor changes in adhesive cell numbers recorded in an aging time/culturing time-dependent manner. Both zirconia showed comparable biological performance before and after aging.
[55]	Digital Light Processing (DLP) (*INNI-II*) Subtractive Manufacturing (SM) (*5X-500L*)	DLP: ZrO_2_ (*INNI-Cera*, *AON*, *Gunpo*) SM: ZrO_2_ (*Luxen Zirconia 1200 Zr*, *Dentalmax*)	Copings	Shrinkage, accuracy, bond strength	DLP led to higher thermal shrinkage and lowest accuracy than SM samples. DLP led to higher bond strength (35.12 ± 4.09 MPa) than SM samples (30.26 ± 5.20 MPa). All samples showed typical adhesive failure mode, showing debonding between the porcelain and zirconia.
[56]	Digital Light Processing (DLP)	3Y-TZP (40 vol%) (*JA-TZP-3Y*; *Jin’ao*)	Crowns	Dimensional accuracy	DLP crowns presented internal fit and marginal adaptation close to clinical standards (239.3 ± 7.9 µm and 128.1 ± 7.1 µm, respectively). SM crowns led to more suitable values for clinical application: 68.5 ± 3.9 µm for internal fit and 71.6 ± 2.8 µm for marginal adaptation.
[57]	Digital Light Processing (DLP) (*ADMAFLEX 130*, *ADMATEC Europe BV*)	3Y-TZP (40.5 vol% and 43.6 vol%) (*CY3Z*, *Saint-Gobain ZirPro*)	Implants	Density, microstructure, flexural strength, elastic modulus, hardness	Relative density of 99.2%TD, regardless of solid loading and printing direction. Homogeneous and defect-free cross section. Flexural strength influenced by printing direction and zirconia vol%: − the higher solid loading (43.6 vol%) led to highest value (751 ± 83 MPa) when tested perpendicular to the printing plane. Elastic modulus (≈160 GPa) and hardness (≈1200 HV) not dependent on the printing direction.
[58]	Digital Light Processing (DLP) (*Cerafab S65*^®^, *Lithoz*) Subtractive Manufacturing (SM) (*DWX-52D*^®^, *DGShape*, *Roland Company*)	DLP: 3Y-TZP (*LithaCon 3Y 210*^®^, *Lithoz*) SM: 3Y-TZP (*Cerafab S65*^®^, *Lithoz*)	Not specified	Microstructure, deformation under compression	DLP surface presented small surface pores (≈3 μm). Some DLP samples reached failure, whereas all the SM samples did not reach failure at the limit of the load cell (1200 MPa). DLP samples showed lower tendency to deformation under compression (11.9 ± 0.1%) than SM samples (13.5 ± 0.5%).
[59]	Digital Light Processing (DLP) (*Octave Light R1*, *Octave Light*)	3Y-TZP (52, 54, 56 vol%) (*TZ-3Y*, *Tosoh*)	Not specified	Density, strength, hardness	Density increased with the ZrO_2_ vol% (94.89 ± 0.35, 95.65 ± 0.67, 96.15 ± 0.59% TD, for 52, 54, and 56 vol%, respectively). The addition of silane coupling agent to the suspension of 56 vol% led to higher strength and hardness (5–6%) compared to those without silane coupling agent.

**Table 2:** Summary of the main results obtained for ZrO_2_-based samples produced by stereolithography (SLA).

Reference	Manufacturing Technology	Ceramic Material	Dental Application	Studied Properties	Main Results
[60]	Stereolithography (SLA) (*SPS450B*, *Shaanxi Hengtong Intelligent Machine*)	3Y-TZP (40 vol%) (*Shanghai Chigong*)	Bridges	Density, surface roughness, hardness, flexural strength	Relative density of 98.58% TD. Surface roughness of 2.06 µm. Hardness of 1398 HV. Flexural strength of 200.14 MPa.
[61]	Stereolithography (SLA)	3Y-TZP	Bridges and implants	Microstructure	Cracks on the outer surface, with a certain propagation orientation. Pores with 200–400 nm distributed all over the surface.
[62]	Stereolithography (SLA) (*CERAMAKER 900*; *3DCeram*) Subtractive Manufacturing (SM) (*DWX-50*; *Roland DG Corp*)	SLA: ZrO_2_ (*3DMIXZrO2L*; *3DCeram*) *SM: ZrO_2_* (*Zenostar*; *Wieland Dental*)	Crowns	Trueness	The trueness of the external surface, intaglio surface, marginal area, and occlusal surface of SLA crowns was similar to that of SM crowns.
[63]	Stereolithography (SLA) (*CSL150*; *PORIMY*)	ZrO_2_ (45 Vol%)	Crowns	Accuracy, flexural strength	Internal fit and marginal adaptation not ideal for clinical application: cement space of 63.4 µm in the occlusal area, 135.1 µm in the axial area, and 169.6 µm in the marginal area. Flexural strength of 812 ± 128 MPa.
[35]	Stereolithography (SLA) (*CeraMaker 900*; *3DCeram*) Subtractive Manufacturing (SM) (*Isomet VR1000 Precision Saw*; *Buehler*)	SLA: 3Y-TZP (*3DMix ZrO_2_*; *3DCeram*) SM: 3Y-TZP (*IPS e.max ZirCAD*; *Ivoclar Vivadent AG*)	Not specified	Density, hardness, flexural strength	SLA samples’ relative density of 98.51% TD. SLA samples’ hardness of 1285 HV. Decrease in flexural strength for SLA and SM samples after artificial ageing treatment: − SLA: 320 ± 41 MPa (before) to 281 ± 39 MPa − (after) − SM: 915 ± 68 MPa (before) to 573 ± 43 MPa (after)
[64]	Stereolithography (SLA) Subtractive Manufacturing (SM)	SLA: 3Y-TZP (*LithaCon 3Y 230*, *Lithoz*; *3D Mix ZrO_2_*, *3DCeram*) and ATZ (*3D Mix ATZ*, *3DCeram*) SM: 3Y-TZP (*LAVA Plus*, *3M Oral Care*).	Implants	Microstructure, flexural strength	3Y-TZP SLA samples revealed a crystal structure, flexural strength, and microstructure similar to SM samples. ATZ SLA samples showed higher flexural strength than 3Y-TZP produced by SLA and SM.
[36]	Stereolithography (SLA) (*CeraFab System S65 Medical*; *Lithoz GmbH*) Subtractive Manufacturing (SM) (*DGShape DWX 52DC*)	SLA: 3Y-TZP (*LithaCon 3Y 210*; *CeraFab System S65 Medical*) SM: 3Y-TZP (*Priti multidisc ZrO2 monochrome*)	Not specified	Microstructure, flexural strength	SLA samples presented a layer strand texture with a smooth depression between the layers (less than 5 and 10 µm). SLA samples showed irregular surface with pits of varying dimensions (10–40 µm), but no evidence of cracks, fracture surfaces, or flaws. SLA samples presented higher flexural strength (1519 ± 254 MPa) than SM samples (981 ± 130 MPa).
[65]	Stereolithography (SLA) (*CSL 100*, *Porimy 3D printing Technology*) Subtractive Manufacturing (SM)	SLA: YSZ (84 wt%–48 vol%) SM: ZrO_2_ (*D98-20*, *Upcera*)	Not specified	Dimensional accuracy, translucency, mechanical properties, microstructure	SLA samples presented dimensional accuracy, translucency and mechanical properties that vary in different build orientations: − Printing in an upright way led to higher relative density (95.4% TD) and translucency (4.393%) than when printed horizontally (94.6% TD and 3.403%, respectively). − Horizontally printed samples led to excellent accuracy and mechanical properties. SLA samples showed stress and weak bonding strength among the successive layers. SLA samples presented internal flaws (pores and agglomerations). SLA samples showed two types of fracture modes: fracture due to stress concentration and splintering due to crack deflection.
[37]	Stereolithography (SLA) (*CeraMaker 900*; *3DCeram*)	3Y-TZP (*3DMix ZrO_2_*, *3DCeram*) ATZ (20 wt% Al_2_O_3_ + 80% wt% ZrO_2_) (*3DMix ATZ*, *3DCeram*)	Abutments and crowns	Fracture resistance	3Y-TZP and ATZ crowns showed similar fracture resistance (1243.5 ± 265.5 N and (1209 ± 204.5 N, respectively). Both crowns fractured at the implant–abutment interface.
[66]	Stereolithography (SLA) (*CSL 100*, *Porimy*) Subtractive Manufacturing (SM) (*AK-D4*, *Aidite*)	SLA: 3Y-TZP (47 vol%) (*DLP1080E*, *Han’s Laser*) SM: PSZ (*SHT*, *Aidite*)	Crowns	Finish line designs evaluation	SLA crowns exhibited margins of rounded line angle and without small flaws, although large chippings were found in knife-edged crowns. SM crowns showed margins of sharp line angle and with separate chippings.
[67]	Stereolithography (SLA) (*CeraBuilder 100*, *Wuhan Intelligent Laser Technology*)	80 wt% 3Y-TZP *(Jiangxi Size Materais*) + 20 wt% Al_2_O_3_ (*Almantis*) (45 vol%)	Implants	Density, hardness, fracture toughness	Relative density of 99.09% TD. Hardness of 1699 HV. Fracture toughness of 6.88 MPa⋅m^1/2^
[38]	Stereolithography (SLA) (*Ceramaker C900 Flex*) Subtractive Manufacturing (SM)	SLA: 3Y-TZP (*3DCeram Co*) SM: 3Y-TZP (*ArgenZ ST*)	Crowns	Microstructure, fracture load, flexural strength, flexural modulus	SLA samples with 0% porosity showed the highest fracture load (1132.7 N), flexural strength (755.1 MPa) and flexural modulus (41.273 GPa). SLA samples with 40% porosity showed the lowest fracture load (72.13 N), flexural strength (48.09 MPa) and flexural modulus (7.177 GPa).
[25]	Stereolithography (SLA) (*3DCeram*) Subtractive Manufacturing (SM)	SLA: 3Y-TZP SM: 3Y-TZP	Crowns	Trueness, precision	SLA crowns revealed the best occlusal trueness (8.77 ± 0.89 µm) and worst intaglio trueness (23.90 ± 1.60 µm). Both SLA and SM crowns presented similar internal fit and marginal adaptation. SLA crowns showed higher precision (9.59 ± 0.75 µm) than SM crowns (17.31 ± 3.39 µm).

**Table 3:** Summary of the main results obtained for ZrO_2_-based samples produced by robocasting (RC).

Reference	Manufacturing Technology	Ceramic Material	Dental Application	Studied Properties	Main Results
[68]	Robocasting (RC)	3Y-TZP (47 vol%) (*Refractron Technologies*)	Not specified	Morphology	Surface with “Stair stepped” appearance. Drying issues (e.g., cracks) observed.
[69]	Robocasting (RC) (*Lulzbot Mini*, *Aleph Objects*) Slip Casting (SC)	RC: 3Y-TZP (80, 82, 84, 86, 88, 90 wt%–44.5, 46.2, 48, 52, 56.4, 61.3 vol%) (*SF YSZ-1011*, *Zircomet*) SC: 3Y-TZP (80 wt%–44.5 vol%) (*SF YSZ-1011*, *Zircomet*)	Not specified	Density, hardness, fracture toughness	Paste with better rheological properties: 56.4 vol% Relative density ≈ 97%TD (for RC and SC). Vickers hardness of 1485 ± 32 HV (RC) and 1397 ± 27 HV (SC). Fracture toughness of 4.11 ± 0.09 MPa.m^1/2^ (RC) and 3.84 ± 0.21 MPa.m^1/2^ (SC).
[70]	Robocasting (RC) (*Delta Wasp 2040 Turbo*, *Wasproject*)	3Y-TZP (60 vol%) (*TZ-3YB-E*, *Tosoh Co*)	Not specified	Density, hardness, fracture toughness, flexural strength	Relative density of 98.1% TD. Hardness of 1175 HV. Fracture toughness of 2.63 MPa.m^1/2^ Flexural strength of 488.96 MPa.
[1]	Robocasting (RC) *(Delta WASP 2040)* Subtractive Manufacturing (SM) (*M5 milling unit*, *Zirkonzahn*)	RC: 3Y-TZP (40 vol%) (*ZPEX*, *Tosoh*) *SM:* 3Y-TZP (*Ice Zirkon Translucent*, *Zirkonzahn*) Glaze (*IPS e.max Ceram Glaze Paste*, *Ivoclar Vivadent*)	Crowns	Density, hardness, fracture toughness, cusp and prosthesis wear	RC samples’ relative density of 98.3% TD. RC samples presented lower hardness (≈1100 HV) and fracture toughness (≈4 MPa.m^1/2^) than SM samples (≈1400 HV and ≈5.2 MPa.m^1/2^, respectively). No wear was found both on RC and SM samples. RC samples induced lower cusp wear. Both RC and SM glazed surfaces and antagonist dental cusps suffered wear, cusps wear being higher than that found for unglazed samples. Cusps tested against RC glazed samples suffered less wear than those opposed to SM glazed samples.
[71]	Robocasting (RC) (*Delta WASP 2040*) Unidirectional Compression (UC)	RC: 3Y-TZP (*TZ-3Y-E*, *Tosoh*) *UC*: 3Y-TZP (*ZPEX*, *Tosoh*)	Crowns	Cusp and prosthesis wear	Both RC and UC samples did not suffer wear. The cusps of RC and UC samples suffered similar wear. RC and UC cusps suffered mild abrasive wear, delamination and fatigue.
[72]	Robocasting (RC) (*Model EBRD-A32*, *3D Inks*)	5Y-PSZ (42 vol%) (*Zpex Smile*,*Tosoh*)	Not specified	Density, hardness, fracture toughness, flexural strength	Relative density ≈ 94% TD. Hardness of 1295 HV. Fracture toughness of 3.91 MPa.m^1/2^. Flexural strength of 285 MPa.

**Table 4:** Summary of the main results obtained for ZrO_2_-based samples produced by material jetting (MJ).

Reference	Manufacturing Technology	Ceramic Material	Dental Application	Studied Properties	Main Results
[73]	Material Jetting (MJ) (*HP Deskjet 930c*^®^) Slip Casting (SC)	MJ: 3Y-TZP (40 vol%) (*TZ-3YS-E*, *Tosoh*) *SC*: 3Y-TZP (40 vol%) (*TZ-3YS-E*, *Tosoh*)	Bridges	Density, microstructure, flexural strength	MJ samples have a relative density of >96%TD. MJ samples revealed a smooth surface without “stair steps” effect and drying or sintering cracks. MJ samples presented higher flexural strength (≈843 MPa) than CS samples (∼684 MPa).
[74]	Material Jetting (MJ)	3Y-TZP (55 vol%) (*ZL-STS-3.0*, *Zhonglong Chemical*)	Crowns	Density, hardness	Relative density of 98.5% TD. Hardness of 1468 HV.
[75]	Material Jetting (MJ)	5Y-TPZ (62.3 wt%–22.5 vol%) (*Sigma-Aldrich*)	Crowns	Density, hardness, fracture toughness	Relative density of 99.5% TD. Hardness of 1516 HV. Fracture toughness of 5.62 MPa.m^1/2^.
[76]	Material Jetting (MJ) (*XJET Carmel 1400 inkjet printer*)	3Y-TZP (45 wt%–12.5 vol%) (*C800 zirconia model dispersion grade 7250001*, *XJET*)	Not specified	Density, microstructure, hardness, fracture toughness, elastic modulus	Density of 99.7% TD. Presence of delamination cracks, agglomerates, spherical pores. Hardness (≈1285 HV) and fracture toughness (≈3.85 MPa.m^1/2^) independent of printing direction. Flexural strength of 1004 ± 138 MPa for samples printed in 0° orientation (most favorable printing direction due to layer buildup and since defects are perpendicular to the applied stress). Elastic modulus higher when printed in 45° orientation (209 ± 5 MPa) than in 0° orientation (206 ± 5 MPa). Both values indicate the presence of defects.

**Table 5:** Summary of the main results obtained for ZrO_2_-based samples produced by different 3D printing techniques.

Reference	Manufacturing Technology	Ceramic Material	Dental Application	Studied Properties	Main Results
[77]	Stereolithography (SLA) (*CSL150*; *PORIMY*) Digital Light Processing (DLP) (*Cerafab 7500*; *Lithoz*) Subtractive Manufacturing (SM) (*DWX-52DCi*; *Roland*)	SLA: ZrO_2_ (50 vol%) (*BLM-FTC-1*; *PORIMY*) *DLP*: *ZrO_2_* (*LithaCon 3Y 230 D*; *Lithoz*) SM: ZrO_2_ (*ST*; *Upcera*)	Not specified	Phase composition, microstructure, flexural strength, before and after ageing	The *m*-phase content increased with the aging time, both for DLP and SLA samples. DLP samples showed zirconia grain fragments, while SLA samples presented grain pullout. Surface defects were not obvious for SM samples. SLA samples presented the highest flexural strength after 5 h-ageing (1010.3 MPa), followed by 10 h-ageing (913.06 MPa) and 15 h-ageing (814.28 MPa). The flexural strength for SM samples was always more than 1200 MPa, and for DLP was ≈800 MPa before and after aging for 5 h, 10 h and 15 h.
[78]	Digital Light Processing (DLP) (*Octave Light R1*; *Octave Light Limited*) Stereolithography (SLA) (*C100 EASY FAB*; *3D Ceram*) Subtractive Manufacturing (SM) (*Dental Designer*; *3Shape*)	SLA: 3Y-TZP (*3D Ceram*) DLP: 3Y-TZP (*M.O.P*) SM: 4Y-PSZ/5Y-PSZ (*KATANA UTML*, *Kuraray Noritake Dental*)	Crowns	Trueness, antagonist wear, microstructure	Similar trueness of intaglio crown surfaces, regardless of the manufacturing method. Similar volume loss of the antagonist teeth: − 2.06 ± 1.24 mm^3^ (DLP) − 1.74 ± 1.20 mm^3^ (SLA) − 2.51 ± 2.13 mm^3^ and 2.40 ± 1.66 mm^3^ (SM-4YZ and SM-5YZ, respectively) All samples showed highly dense structures with no pores or other manufacturing defects, showing similar morphologies of fractured surfaces.
[79]	Stereolithography (SLA) (*Ceramaker900*, *3DCeram*) Material Jetting (MJ) (*XJET*, *Rehovot*) Digital Light Processing (DLP) (*DLP_1_*: *405 nm Prototype DLP-Printer*; *DLP_2_*: *CeraFab System Medical*, *Lithoz*) Subtractive Manufacturing (SM)	SLA: 3Y-TZP (*3D Mix ZrO2 Zr-P03 grade*, *3DCeram*) MJ: 3Y-TZP (*C800 zirconia model dispersion grade 7250001*, *XJET*) DLP_1_: 3Y-TZP (*prototype printer*) DLP_2_: (*LithaCon 3Y 230*, *Lithoz*) SM: 3Y-TZP (*Zirconia ST*, *GC* (*s_1_*); *ZrO_2_* “*translucent*” *Pritidenta* (*s_2_*))	Not specified	Accuracy, surface deviation	SM led to the most accurate samples with no significant difference regarding the material. SM only led to differences on accuracy relative to samples produced by with MJ and SLA for s_1_ material. Mean surface deviation <50 µm for samples produced by SM and MJ and <100 µm for SLA and DLP_2_. DLP_1_ showed surface deviations >100 µm, leading to the least accurate samples.
[80]	3D Gel Deposition (3DGD) Cold Isostatic Pressing (CIP)	Self-glazed zirconia (SG) Conventional zirconia (CZ)	Not specified	Microstructure, fracture force before and after fatigue tests	SG presented a fine-grained microstructure with no visible microscopic voids. Fracture force of SG significantly higher (≈8000 N) than that of CZ (≈7000 N), both before and after fatigue tests (no statistically significant difference). Both SG and CZ showed slightly increased fracture force after fatigue tests due to the stability reduction of the tetragonal phase by fatigue stress. Both SG and CZ withstood occlusal forces applied in the posterior region. SG more suitable to be used in the anterior regions due to aesthetic reasons (improved optical translucency).

## Data Availability

No new data were created or analyzed in this study. Data sharing is not applicable to this article.
